# Haemothorax after removal of subclavian venous catheter: An unusual complication

**DOI:** 10.4103/0019-5049.72661

**Published:** 2010

**Authors:** Srinivasan Swaminathan, Rajnish K Jain

**Affiliations:** Department of Anaesthesiology and Critical Care, Bhopal Memorial Hospital and Research Centre, Bhopal, India

**Figure 1:**
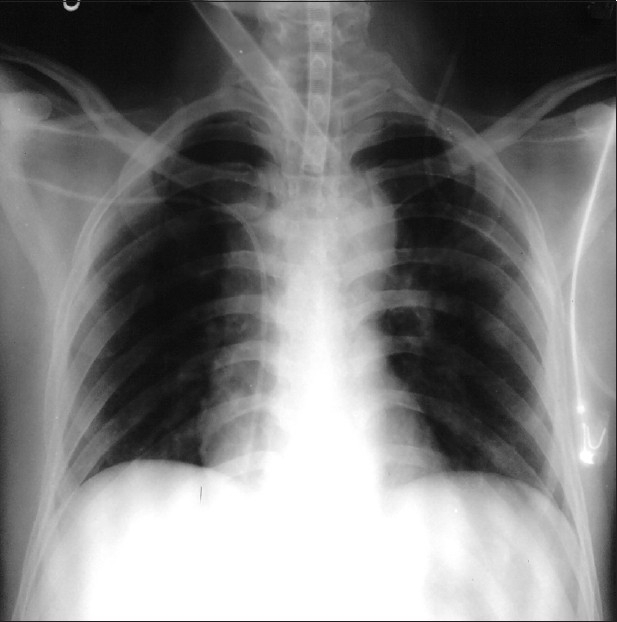
Chest X-ray with catheter *in situ*

Sir,

We like to report a case of haemothorax which occurred after removal of subclavian venous catheter. A 35 year old male patient, a case of left Cerebello pontine angle tumour was posted for craniectomy and excision. On the day of surgery after induction of general anaesthesia, subclavian venous catheterization was done on the right side through standard infraclavicular approach. Central venous pressure monitoring was done intraoperatively and intraoperative haemodynamics and vital parameters remained normal. After surgery patient was shifted to the intensive care unit for postoperative ventilatory support. Chest X-ray taken in the postoperative period with the catheter *in situ* was normal [Figure 1].

In the ICU, patient was weaned from ventilator support and extubated on the first postoperative day. Patient was conscious and oriented and was maintaining stable haemodynamics after extubation.

**Figure 2:**
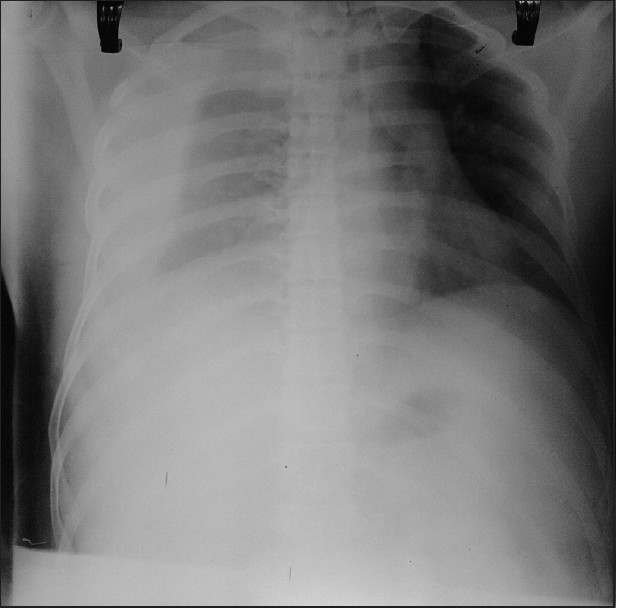
Chest X-ray, haemothorax

**Figure 3:**
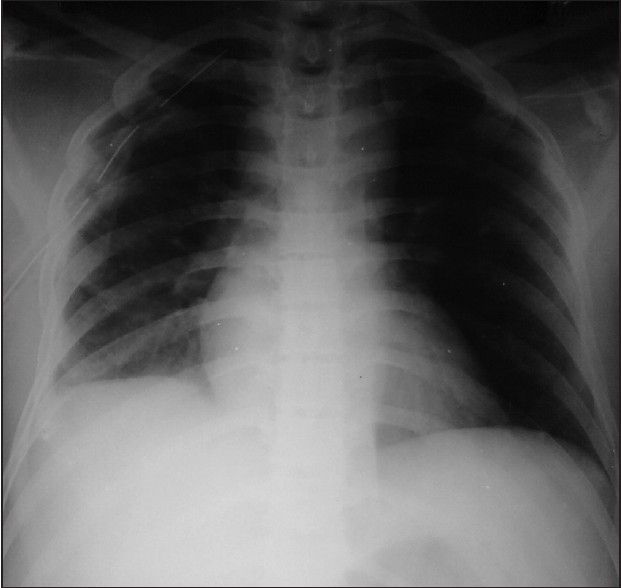
Chest X-ray with ICD

On the third postoperative day subclavian catheter was removed and dressing applied. Within 2 h after removal of catheter, patient started complaining of respiratory difficulty with pain on right side of chest. Patient was maintaining an SpO_2_ of 91–93% and clinical examination revealed reduced air entry on the right side of the chest. Chest X-ray taken showed effusion on right chest with collapse of right lung [Figure 2]. Intercostal drain insertion was done in the right side of the chest and about 1 litre of blood got collected in the ICD bag. Flow of blood through ICD gradually got reduced and stopped after a few hours [Figure 3]. Patient became comfortable with stable vitals after ICD insertion. ICD was retained for three days and then removed. Repeat chest X-rays showed no further collections and the patient was discharged.

Various complications like pneumothorax and haemothorax have been reported to occur during insertion of subclavian venous catheter.[1,2] But, haemothorax occurring after subclavian catheter removal is an unusual complication. Massive haemothorax after subclavian catheter removal in a patient who had undergone renal transplant was reported previously by Collini in 2002.[3] The probable mechanism behind this complication could be injury to the pleura during insertion and a communication could have occured between the vein and right pleural cavity after catheter removal. This complication has been reported to emphasize that careful monitoring is necessary after subclavian venous catheter removal.
